# Recovery Rate of Under‐Five Children From Severe Acute Malnutrition and Its Predictors in Ethiopia: A Systematic Review and Meta‐Analysis

**DOI:** 10.1002/hsr2.71788

**Published:** 2026-01-26

**Authors:** Temesgen Gebeyehu Wondmeneh, Oumer Abdulkadir Ebrahim

**Affiliations:** ^1^ Department of Public Health College of Medical and Health Science Samara University Semera Ethiopia

**Keywords:** Ethiopia, recovery rate, severe acute malnutrition, under‐five children

## Abstract

**Background:**

Previous reviews on severe acute malnutrition recovery in Ethiopia were limited by outdated data and narrow scope. This study used recent and regionally diverse data, including from conflict‐affected areas, to inform current nutrition efforts.

**Objective:**

To provide up‐to‐date information on the recovery rate of Children under five from severe acute malnutrition in Ethiopia.

**Methods:**

A systematic review and meta‐analysis of observational studies published between January 2015 and July 2025 was conducted. PubMed, Scopus, Web of Science, Google Scholar, and African Journals Online were searched. The pooled prevalence was estimated using random‐effects model with 95% confidence intervals. Publication bias was assessed using funnel plots and Egger's test.

**Results:**

The pooled recovery rate of under‐five children from severe acute malnutrition was 71.37% (95% CI: 67.57–75.16%). Anemia (AHR = 0.75, 95% CI: 0.63–0.87), shock (AHR = 0.52, 95% CI: 0.13–0.92), HIV/AIDS (AHR = 0.71, 95% CI: 0.44–0.97), use of a nasogastric tube (AHR = 0.62, 95% CI: 0.31–0.93), vomiting (AHR = 0.65, 95% CI: 0.43–0.86), diarrhea (AHR = 0.71, 95% CI: 0.56–0.86), tuberculosis (AHR = 0.47, 95% CI: 0.34–0.60), and pneumonia (AHR = 0.73, 95% CI: 0.61–0.84) were associated with decreased recovery rate. Conversely, the use of amoxicillin (AHR = 2.10, 95% CI: 1.54–2.70), vitamin A supplementation (AHR = 1.50, 95% CI: 1.21–1.79), absence of malaria (AHR = 1.51, 95% CI: 1.03–1.98), deworming (AHR = 1.45, 95% CI: 1.04–1.82), and provision of ready‐to‐use therapeutic food (AHR = 1.63, 95% CI: 1.07–2.20) were associated with improved recovery rate.

**Conclusion:**

The recovery rate of under‐five children from severe acute malnutrition in Ethiopia remained below national and international targets. Strengthening early detection and management of symptoms and comorbidities, along with scaling up proven interventions, is essential to improving recovery rate.

## Summary

- What is Known
  ◦ Previous reviews on severe acute malnutrition (SAM) recovery in Ethiopia relied on older data and lacked coverage of conflict‐affected regions, limiting their relevance to current national nutrition challenges.
- What is New
  ◦ This study synthesizes recent, regionally diverse evidence (2015–2025) and provides an updated pooled recovery rate of 71.37%, identifying clinical factors that significantly decrease or improve recovery among children under five.
- Clinical Implications
  ◦ Strengthening management of comorbid conditions (e.g., anemia, diarrhea, HIV, pneumonia) and ensuring access to amoxicillin, vitamin A, deworming, and ready‐to‐use therapeutic foods can improve SAM recovery outcomes in Ethiopian treatment programs.

**Abbreviations**
AHR: adjusted hazard ratio
CI: confidence interval
Fig: Figure
HIV/AIDS: Human immunodeficiency virus/acquired immunodeficiency syndrome
NG tube: nasogastric tube
RUTF: Ready use therapeutic food
S: Supplementary
TB: Tuberculosis

## 1 Introduction

Acute malnutrition is a condition caused by insufficient intake of energy and/or protein, typically due to poor diet or disease, and is characterized by rapid weight loss or the presence of nutritional edema [1, 2]. Severe acute malnutrition (SAM) is characterized by severe wasting, defined as a weight‐for‐height/length below −3 standard deviations (SD) of the WHO growth standards, a mid‐upper arm circumference (MUAC) of less than 115 mm, and/or the presence of bilateral pitting edema in both feet [1, 3]. As of 2024, undernutrition remains a major global health concern among children under the age of five, with 150.2 million (23.2%) stunted—primarily in Asia (51%) and Africa (43%)—and 42.8 million wasted, including 12.2 million (1.9%) affected by severe wasting [4]. Despite declines in case fatality rates, children with severe acute malnutrition (SAM) still have low recovery rates globally, ranging from 22% to over 90% depending on context [5]. According to the sphere standard, an acceptable level of recovery should be above 75% [6]. However, these standards have not yet been met in developing countries [7]. Recovery rates for children with severe acute malnutrition were 68.6% in Pakistan [8], 78% in a community‐based intervention in India [9], and 46.4% in a multicenter study conducted across South Asia [10]. In sub‐Saharan Africa, the recovery rate of children from severe acute malnutrition was 71.2% [11]. In Nigeria, the recovery rate of children aged 6–59 months with complicated severe acute malnutrition was 95.7% [12]. In Ethiopia, the recovery rate of children from severe acute malnutrition ranged from 70% to 72% [13, 14, 15].

Previous studies have shown that the absence of comorbid conditions such as edema, hypothermia, pneumonia, anemia, and HIV was associated with improved recovery outcomes among children suffering from severe acute malnutrition [16, 17, 18, 19]. Severely malnourished children co‐infected with tuberculosis (TB) have been found to have a higher risk of mortality than those without TB [20]. The presence of pneumonia and edema in children with severe acute malnutrition has been associated with lower nutritional recovery rates. Additionally, children with diarrhea tend to experience longer recovery times compared to those without diarrhea [21]. Children receiving ready‐to‐use supplementary foods demonstrated significant improvements in weight, height, and body mass index [22]. Furthermore, children experiencing mild to moderate food insecurity exhibited faster recovery rates from severe acute malnutrition compared to those facing severe food insecurity [23]. The routine use of antibiotics, such as amoxicillin, has also been shown to enhance nutritional recovery in children with uncomplicated severe acute malnutrition [24, 25].

The burden of malnutrition among children under five remains high globally [26, 27]. According to a recent UNICEF report in Ethiopia, 38.6% of children under the age of five were stunted, 21% were underweight, and 7% were wasted, reflecting persistent malnutrition [28]. Previous systematic reviews and meta‐analyses have assessed the recovery rate from severe acute malnutrition (SAM) among under‐five children in Ethiopia, but they are limited by outdated data, narrow geographic coverage, and a lack of focus on the most recent conflict‐affected settings. Most did not incorporate recent studies conducted after the onset of major internal conflicts that have disrupted health services and food systems in Ethiopia. Our study addresses these gaps by including the most recent and regionally diverse data, with particular attention to conflict‐affected areas, to provide an updated and nationally representative estimate of recovery rates and their predictors. This evidence is critical for guiding post‐conflict nutrition strategies and improving SAM management in Ethiopia.

## 2 Materials and Methods

### 2.1 Protocol Registration and Study Design

The study protocol was registered in the PROSPERO database under the registration number CRD42024526856. A systematic review and meta‐analysis of observational studies published between January 1, 2015, and July 23, 2025, was conducted. The findings were reported in accordance with the Preferred Reporting Items for Systematic Reviews and Meta‐Analyses Protocols (PRISMA‐P) guidelines [29] (Supporting file 1).

### 2.2 Searching Strategies

A comprehensive search was conducted to identify relevant studies on the recovery rate of children under 5 years of age from severe acute malnutrition (SAM) in Ethiopia. Studies published in English were retrieved from reputable databases, including Web of Science, Scopus, PubMed, Google Scholar, and African Journals Online (AJOL). To minimize the risk of missing eligible studies, additional sources were explored through manual searching of reference lists and hand‐searching of relevant journals. Two reviewers (Temesgen Gebeyehu Wondmeneh (TGW) and Oumer Abdulkadir Ebrahim (OAE)) independently conducted the search using predefined key terms. The search strategy was guided by four main components: children (population), severe acute malnutrition (condition), recovery (outcome), and Ethiopia (context). Boolean operators such as “AND” and “OR” were used to appropriately combine the search terms. Prior to the full search, the suitability of the selected keywords was assessed through a preliminary search in PubMed. The literature search was limited to studies published between January 1, 2015, and July 23, 2025, to ensure the inclusion of recent and relevant evidence. The initial electronic search was conducted from July 3 to 10, 2024, and was subsequently updated from July 21 to 23, 2025 (Supporting File 2).

### 2.3 Study Selection

All articles initially identified through the search strategy were exported to EndNote version 8, and duplicates were removed. The titles and abstracts of the remaining articles were independently screened by two authors (TGW and OAE), and irrelevant studies were excluded. Full‐text versions of the selected articles were then retrieved and thoroughly reviewed to assess their eligibility.

### 2.4 Eligibility Criteria

The PICO framework was used to define the inclusion criteria, focusing on Population (under‐five children), Condition (severe acute malnutrition), Outcome (recovery), and Context (Ethiopia), summarized as PCOCo. Original research articles employing cross‐sectional or cohort study designs that reported the recovery rate of children from severe acute malnutrition were considered for inclusion. In contrast, review articles, studies lacking full‐text access after contacting the corresponding author, studies reporting outcomes for children over 5 years of age, and studies without clearly defined recovery outcomes were excluded.

### 2.5 Outcome Measures

The primary outcome of this review was the pooled recovery rate of under‐five children from severe acute malnutrition (SAM) in Ethiopia. Studies were included if they reported either the percentage of under‐five children who recovered from SAM or the number of recovered cases along with the total sample size, allowing for the calculation of a pooled recovery rate. The secondary outcome was to identify predictors associated with recovery from SAM. For this purpose, adjusted hazard ratios (AHRs) reported in the primary studies were extracted and analyzed for dichotomous variables related to recovery outcomes.

### 2.6 Research Questions

1. What is the pooled recovery rate of under‐five children from severe acute malnutrition in Ethiopia?
2. What are the predictors for the recovery rate of under‐five children from severe acute malnutrition in Ethiopia?

### 2.7 Data Extraction

Data were extracted using a predefined data extraction format developed by two authors (TGW and OAE) in Microsoft Excel. Any disagreements between the authors were resolved through scientific consensus. Extracted data included the first author's name, year of publication, study region, study design, sample size, number of children recovered from severe acute malnutrition, percentage of recovered children, and median time to recovery (Supporting File 3).

### 2.8 Quality of Included Studies

The risk of bias for each included study was independently assessed by two authors (TGW and OAE) using the Newcastle‐Ottawa Scale (NOS) for cohort studies [30]. Any disagreements were resolved through scientific consensus. The NOS evaluates study quality across three domains: selection, comparability, and outcome. The selection domain includes four items: representativeness of the exposed cohort, selection of the non‐exposed cohort, ascertainment of exposure, and confirmation that the outcome of interest was not present at the start of the study. The outcome domain includes assessment of the outcome, adequacy of the follow‐up period, and completeness of follow‐up. The comparability domain includes a single item assessing whether the study controlled for confounding factors. Each item in the selection and outcome domains can receive a maximum of one star, while the comparability domain allows for up to two stars. The total quality score ranges from 0 to 9. Studies scoring 6–9 were considered to have good quality, 4–5 fair quality, and 0–3 poor quality. Only studies rated as fair to good quality were included in the final analysis to ensure methodological rigor.

### 2.9 Data Synthesis and Analysis

The extracted data in Microsoft Excel were imported into STATA version 15 for analysis. A random‐effects model [31] was employed to estimate the pooled recovery rate of children with severe acute malnutrition. The results of the meta‐analyses were presented using summary tables and forest plots. Pooled estimates of the recovery rate and associated predictors were reported with corresponding 95% confidence intervals (CIs). Heterogeneity among studies was assessed using I² statistic and Cochrane's Q test [32, 33]. I² values of 0%, 25%, 50%, and 75% were interpreted as indicating no, low, moderate, and high heterogeneity, respectively. In this review, heterogeneity was considered significant when I² was ≥ 50% and the *p*‐value was < 0.05. Publication bias was assessed through visual inspection of the funnel plot and Egger's test [34, 35]. When evidence of publication bias was detected, the trim‐and‐fill method was applied to estimate and adjust for the potential impact of missing studies [36]. This method accounts for publication bias by identifying and imputing studies that may be missing due to non‐significant results. Additionally, subgroup analyses and meta‐regression were performed to explore potential sources of heterogeneity. Sensitivity analysis was conducted to determine whether the pooled recovery rate was unduly influenced by any single study.

### 2.10 Ethical Approval

This systematic review used previously published studies and did not involve human participants; therefore, ethical approval was not required.

## 3 Results

### 3.1 Study Selection

**Figure 1:**
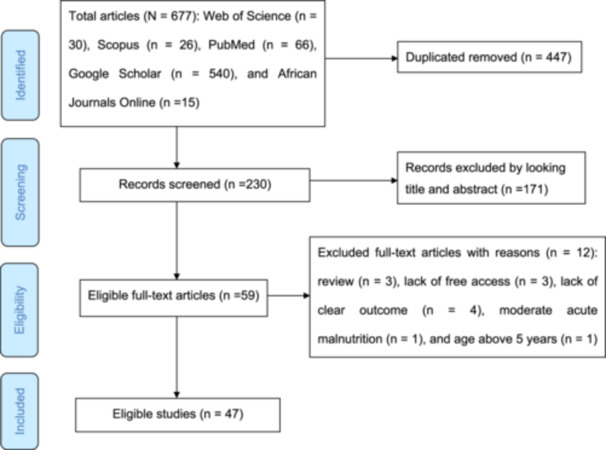
Shows the PRISMA flow chart for the selection of studies for systematic review.

The initial electronic database search yielded a total of 677 articles from Web of Science (*n* = 30), Scopus (*n* = 26), PubMed (*n* = 66), Google Scholar (*n* = 540), and African Journals Online (*n* = 15). After removing 447 duplicate records, 230 articles remained. Based on title and abstract screening, 171 irrelevant articles were excluded. The full texts of the remaining 59 articles were reviewed, and 12 were excluded due to being reviews (*n* = 3), lacking free access (*n* = 3), having unclear outcomes (*n* = 4), focusing on moderate acute malnutrition (*n* = 1), or including children over 5 years (*n* = 1). Finally, 47 studies met the eligibility criteria and were included in the systematic review (Figure. 1).

### 3.2 Study Characteristics

This systematic review included a total of 22,192 under‐five children from 47 studies. The majority of the studies (39 out of 47) were retrospective, while the remaining eight were prospective cohort studies. Fourteen studies were conducted in the Amhara region, ten in the Southern Nations Nationalities Peoples' Region (SNNPR), and nine in Oromia. Addis Ababa contributed five studies, while Tigray, Benishangul‐Gumuz, and Afar each had two. Each of the Somali region, Harari, and Dire Dawa administrative city had one study. About 39 studies were conducted in 2019 or later. The smallest and largest sample sizes were 133 [37] and 1690 [38], respectively. The minimum and maximum recovery rates were 25.6% [37] and 95.9% [39], respectively (Table 1).

**Table 1:** Study characteristics.

ID	Authors, publication year	Region	Study design	Sample size	Recovered Children	%
1.	Budul AB, et al [40]. 2020	Somali	Retrospective	350	286	81.7%
2.	Akeberegn A, et al [41]. 2023	SNNP	Retrospective	241	192	79.7%
3.	Negussie AS, et al [42]. 2020	Addis Ababa	Retrospective	304	214	70.4%
4.	Baraki AG, et al [38]. 2020	Amhara	Retrospective	1690	1050	62.13%
5.	Bekalu A, et al [23]. 2022	Oromia	Prospective	423	327	77.3%
6.	Wondim A, et al [43]. 2020	Benishangul Gumuz	Retrospective	398	262	65.8%
7.	Fikrie A, et al [44]. 2019	SNNP	Retrospective	381	272	69.3%
8.	Abebe A, et al [45]. 2023	SNNP	Prospective	476	340	71.4%
9.	Tsegaye A, et al [7]. 2022	Oromia	Retrospective	357	284	79.6%
10.	Tegegne AS, et al [46]. 2021	Afar	Retrospective	650	408	62.9%
11.	Derseh, B, et al [19]. 2018	Amhara	Retrospective	413	231	55.9%
12.	Lencha B, et al [47]. 2023	Oromia	Retrospective	763	711	93.2%
13.	Atnafe B, et al [48]. 2019	Dire Dawa	Retrospective	713	569	79.8%
14.	Abate BB, et al [49]. 2020	Amhara	Retrospective	600	390	65%
15.	Asres DT, et al [50]. 2018	Amhara	Retrospective	401	208	51.9%
16.	Gebremichael DY [51]. 2015	SNNP	Retrospective	420	346	82.4%
17.	Gebrezgi D, et al [52]. 2019	Amhara	Retrospective	401	208	51.9%
18.	Wagnew F, et al [53]. 2019	Amhara	Retrospective	416	288	69.2%
19.	Bizuneh FK, et al [54]. 2022	Benishangul Gumuz	Retrospective	454	297	65.4%
20.	Yadeta SK, et al [55]. 2024	Oromia	Retrospective	402	360	89.6%
21.	F Adem, et al [37]. 2020	Oromia	Prospective	133	34	25.6%
22.	Kidane GF, et al [56]. 2023	Tigray	Prospective	232	176	75.9%
23.	Teshome G, et al [57]. 2019	SNNP	Prospective	216	172	79.6%
24.	Mekuria G, et al [58]. 2017	Amhara	Retrospective	253	197	77.9%
25.	Kitesa GY, et al [59]. 2023	Oromia	Retrospective	590	471	79.8%
26.	Desyibelew HD, et al [60]. 2017	Amhara	Retrospective	401	234	58.4%
27.	Gebremedhin K, et al [61]. 2020	SNNP	Retrospective	402	283	70.4%
28.	Adimasu M, et al [62]. 2020	Addis Ababa	Retrospective	423	344	81.3%
29.	Mengesha MM, et al [63]. 2016	SNNP	Retrospective	348	274	78.75
30.	Kabalo MY, et al [64]. 2017	SNNP	Retrospective	794	504	64.9%
31.	Shanka NA, et al [65]. 2015	SNNP	Retrospective	771	522	67.7%
32.	Kabthymer RH, et al [66]. 2020	Oromia	Retrospective	375	274	73.15
33.	Hassen SL, et al [67]. 2019	Amhara	Retrospective	406	306	75.4%
34.	Eyi SE, et al [68]. 2022	Oromia	Retrospective	486	334	68.7%
25.	Husen S, et al [69]. 2022	Oromia	Retrospective	1004	913	90.9%
36.	Tefera TK, et al [70]. 2020	Amhara	Prospective	341	254	74.5%
37.	Tesfay W, et al [71]. 2020	Tigray	Retrospective	564	316	56%
38.	Mamo WN, et al [72]. 2019	Amhara	Prospective	389	254	65.3%
39.	Simachew Y, et al [73]. 2020	SNNP	Retrospective	554	390	70.4%
40.	Bitew ZW, et al [74]. 2020	Addis Ababa	Retrospective	515	407	79%
41.	Bitew ZW, et al [75]. 2021	Addis Ababa	Retrospective	610	466	74.6%
42.	Wondmeneh TG, et al [76]. 2025	Afar	Retrospective	372	216	58.1%
43.	Workie H.M, et al [39]. 2025	Amhara	Retrospective	389	372	95.9%
44.	Meseret F, et at [77]. 2024	Harar	Retrospective	349	290	83.1%
45.	Getahun GK, et al [78]. 2024	Addis Ababa	Retrospective	461	222	48.2%
46.	Mekonnen GB, et al [79]. 2025	Amhara	Retrospective	209	157	75.1%
47.	Feleke FW, et al [80]. 2024	Amhara	Prospective	352	263	74.7%

Abbreviation: SNN, Southern Nation Nationality People.

### 3.3 Quality of Included Studies

The methodological quality of the included cohort studies was independently assessed by two authors (TGW and OAE) using the Newcastle‐Ottawa Scale. Based on the appraisal, six studies scored 7 out of 9, five studies scored 8 out of 9, and the remaining 36 studies achieved a full score of 9 out of 9. All studies were considered to be of good quality and were included in the final meta‐analysis 4 (Supporting file 4).

**Figure 2:**
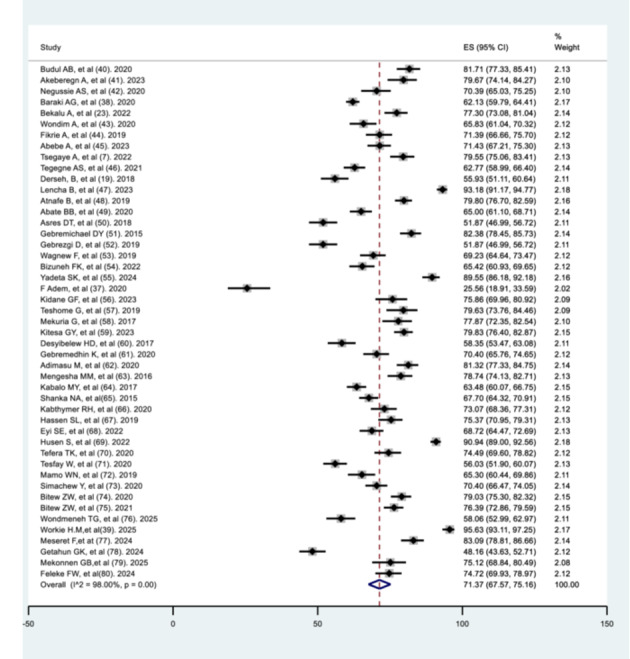
Pooled recovery rate of children from severe acute malnutrition in Ethiopia.

**The pooled recovery rate of under‐five children from severe acute malnutrition** Using a random‐effects model meta‐analysis, the pooled recovery rate of children from severe acute malnutrition in Ethiopia was 71.37% (95% CI: 67.57–75.16%). There was substantial heterogeneity across studies (I² = 98%, *p* < 0.001) (Figure 2).

### 3.4 Subgroup Analysis

We conducted subgroup analyses based on publication year, region, study design, sample size, median recovery time, and clinical classification of SAM. The highest recovery rates from severe acute malnutrition were found in the Harari, Somali, Dire Dawa, Oromia, and SNNP regions, with rates of 83.1%, 81.7%, 79.8%, 75.8%, and 73.4%, respectively. The lowest recovery rates from severe acute malnutrition were found in the Afar, Tigray, Benishangul‐Gumuz, and Amhara regions, with rates of 61%, 63.1%, 65.6%, and 68.1%, respectively. The recovery rate based on retrospective studies was 72% (95% CI: 67.8–76.2). For studies published between 2015 and 2018, the recovery rate was 68.5% (95% CI: 61.3–75.6), while for those published between 2019 and 2025, it was 72.1% (95% CI: 67.8–76.3). In studies with a sample size greater than 420, the recovery rate was 72.9% (95% CI: 67.1–78.7). About 68.1% of children recovered within a median time of less than 1 month (95% CI: 62.9–73.3). Based on the clinical classification of SAM, children with complicated SAM had a recovery rate of 70.5% (95% CI: 66.5–74.6) (Table 2).

**Table 2:** Subgroup analysis.

Variables	Categories	Number of included studies	Recovery rate (95% CI)	Heterogeneity (I^2^), *p*‐value
Region	SNNP	10	73.4% (69.4–77.5)	89.9%, < 0.001
	Amhara	14	68.1% (59.8–76.4)	98.4%, < 0.001
	Oromia	9	75.8% (67.8–83.8)	98.4%, < 0.001
	Addis Ababa	5	71.1% (60.3–81.9)	97.3%, 0.01
	Tigray	2	63.1% (59.8–66.4)	—
	Benishangul Gumuz	2	65.6% (62.4–68.8)	—
	Dire Dawa	1	79.8% (76.7–82.6)	—
	Harar	1	83.1% (78.8–86.7)	
	Somali	1	81.7% (77.3–85.4)	—
	Afar	2	61% [58, 59, 60, 61, 62, 63, 64]	—
Study design	Prospective	8	68.3% (59.7–76.9)	96.0%, < 0.001
	Retrospective	39	72% (67.8–76.2)	98.2%, < 0.001
Publication year	2019–2025	38	72.1% (67.8–76.3)	98.1%, < 0.001
	2015–2018	9	68.5% (61.3–75.6)	96.1%, < 0,001
Sample size	> 420	18	71.6% (65.7–77.5)	98.5%, < 0.001
	≤ 420	28	71.2% (66.1–76.3)	97.5%, < 0.001
Median recovery time	Less than 1 month	28	68.1% (62.9–73.3)	97.9%, < 0.001
	Greater than 1 month	13	77.3% (72.3–82.3)	96.3%, < 0.001
Clinical classification of SAM	Complicated	43	70.5% (66.5–74.6)	98.1%, < 0.001
	Uncomplicated	4	80.4% (73.3–87.5)	92.7%, < 0.001

*Note:* Dash (—) indicates no heterogeneity.

Abbreviation: SNN, Southern Nation Nationality People.

Univariate meta‐regression was conducted to assess whether publication year, sample size, and median recovery time explained the observed heterogeneity. None of the variables were statistically significant: publication year (β = 0.012, *p* = 0.099), sample size (β = 0.0003, *p* = 0.64), and median recovery time (β = 0.002, *p* = 0.10) (Table 3).

**Table 3:** Univariate meta‐regression analysis.

Percentage of recovery	Coefficient	*p*‐value
Publication year	0.01	0.81
Sample size	0.0003	0.52
Median recovery time	0.002	0.8

### 3.5 Publication Bias

**Figure 3:**
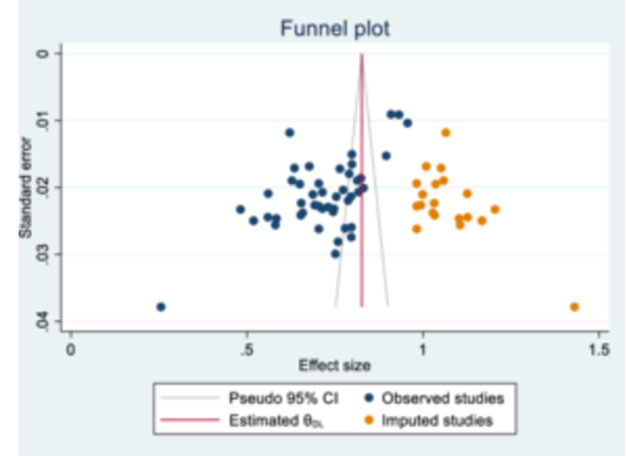
Funnel plot.

In this meta‐analysis, 47 studies were included to estimate the recovery rate among children with severe acute malnutrition (SAM). Publication bias was identified through Egger's test (bias = −14.7; 95% CI: −19.5 to −9.98; *p* < 0.001) and an asymmetric funnel plot. To address this bias, the trim‐and‐fill method was applied, estimating 21 potentially missing studies. The pooled recovery rate based on the observed studies was 71.4% (95% CI: 67.6–75.2), which increased to 82.6% (95% CI: 78.4–86.9) after including the imputed studies. These findings suggest that the true recovery rate may be underestimated in the published literature, likely due to the underreporting of studies with higher recovery outcomes (Figure 3).

### 3.6 Sensitivity Analysis

**Figure 4:**
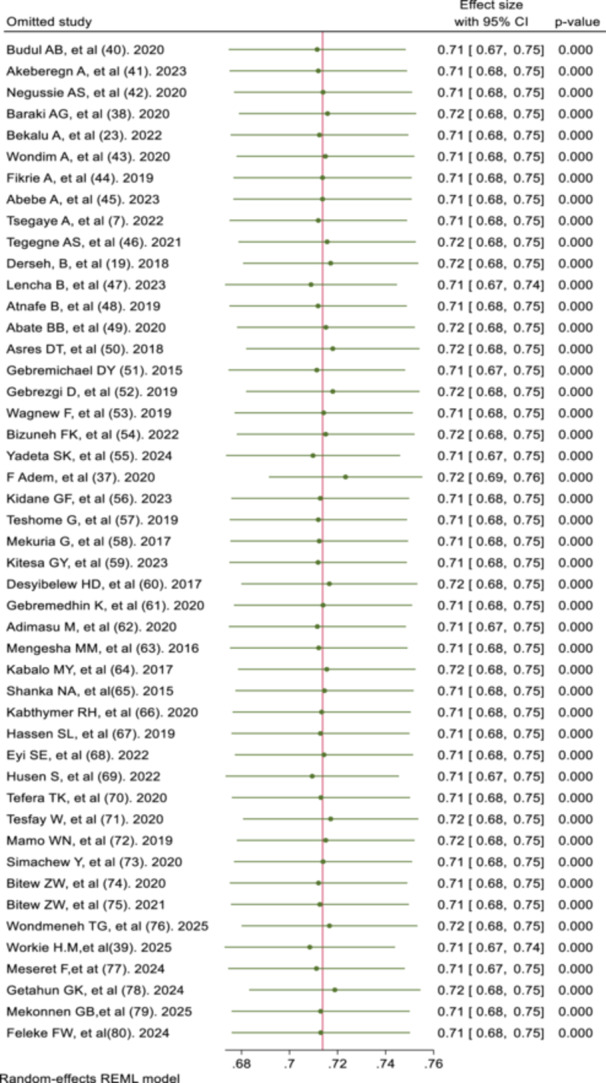
Sensitivity analysis.

The sensitivity analysis demonstrated that the pooled recovery rate of 71.37% remained consistent, with estimates varying narrowly between 71% and 72% upon sequential exclusion of individual studies. This minimal variation indicates the absence of any influential single‐study effect, confirming the robustness and stability of the meta‐analytic estimate. Consequently, the overall recovery rate is not disproportionately affected by any individual study, strengthening the validity of the synthesized result (Figure 4).

**Predictors of recovery of under‐five children from severe acute malnutrition in Ethiopia**

The recovery rate was significantly associated with comorbidities (such as TB, HIV, pneumonia, malaria, and anemia), certain symptoms (including diarrhea, vomiting, and shock), and specific treatments (such as NG tube feeding, vitamin A, ready‐to‐use therapeutic food, amoxicillin, and deworming).

Children diagnosed with TB (AHR = 0.47, 95% CI: 0.34–0.60), HIV (AHR = 0.70, 95% CI: 0.44–0.97), pneumonia (AHR = 0.73, 95% CI: 0.61–0.84), and anemia (AHR = 0.75, 95% CI: 0.63–0.87) experienced 53%, 30%, 27%, and 25% lower recovery rates, respectively, compared to those without these conditions. Children without malaria exhibited a 1.5 times higher recovery rate than those with malaria (AHR = 1.51, 95% CI: 1.03–1.98). Moreover, the presence of symptoms such as diarrhea (AHR = 0.71, 95% CI: 0.56–0.86), vomiting (AHR = 0.65, 95% CI: 0.43–0.86), and shock (AHR = 0.52, 95% CI: 0.13–0.92) clearly demonstrated significantly lower recovery outcomes compared to children without these symptoms. Children who received vitamin A supplementation (AHR = 1.5, 95% CI: 1.2–1.79), ready‐to‐use therapeutic food (AHR = 1.63, 95% CI: 1.07–2.2), amoxicillin (AHR = 2.1, 95% CI: 1.54–2.7), and deworming treatment (AHR = 1.43, 95% CI: 1.04–1.82) had higher recovery rates compared to those who did not receive these treatments. In contrast, children who were treated with NG tube feeding had a lower recovery rate than those who were not (AHR = 0.62, 95% CI: 0.31–0.93) (Table 4).

**Table 4:** Predictors of severe acute malnutrition in Ethiopia.

Predictors	Categories	Number of studies	Pooled AHR (95%CI)	I^2^, *p*‐value
Age	> 24 months	6	1.01 (0.83–1.2)	55.8%, 0.046
	≤ 24 months		1	
Gender	Males	4	1.0 (0.81–1.19)	62.3%, 0.047
	Females		1	
Residence	Urban	8	1.08 (0.95–1.22)	0.0%, 0.47
	Rural		1	
Admission type	New admission	4	1.39 (1.0–1.78)	32.9%, 0.215
	Readmission		1	
Edematous	Yes	5	0.87 (0.61–1.13)	76.4%, 0.002
	No		1	
TB	Yes	7	0.47 (0.34–0.6)*	26.7%, 0.225
	No		1	
HIV	Yes	6	0.71 (0.44–0.97)*	31.4%, 0.2
	No		1	
Pneumonia	Yes	8	0.73 (0.61–0.84)*	40.3%, 0.11
	No		1	
Malaria	No	3	1.51 (1.03–1.98)*	0.0%, 0.73
	Yes		1	
Anemia	Yes	9	0.75 (0.63–0.87)*	54.5%, 0.025
	No		1	
Diarrhea	Yes	12	0.71 (0.56–0.86)*	86.1%, < 0.001
	No		1	
Vomiting	Yes	7	0.65 (0.43–0.86)*	86.7%, < 0.001
	No		1	
Shock	Yes	3	0.52 (0.13–0.92)*	78.3%, 0.01
	No		1	
Fever	Yes	3	0.96 (0.81–1.12)	0.0%, 0.55
	No		1	
NG tube	Yes	3	0.62 (0.31–0.93)*	57.9%, 0.093
	No		1	
Folic acid supplementation	No	7	0.84 (0.59–1.1)	66.5%, 0.006
	Yes		1	
Vitamin A	Yes	6	1.5 (1.21–1.79)*	51.2%, 0.068
	No		1	
Ready used therapeutic food	Yes	3	1.63 (1.07–2.2)*	77.5%, 0.012
	No		1	
Taking amoxicillin	Yes	8	2.1 (1.54–2.7)*	83.6%, < 0.001
	No		1	
Deworming	Yes	7	1.43 (1.04–1.82)*	66.7%, 0.006
	No		1	
Vaccinated with measles	Yes	3	1.07 (0.92–1.21)	0.0%, 0.391
	No		1	

Note:* indicates statistically significant at *p* < 0.05.

## 4 Discussion

Acute malnutrition due to inadequate energy or protein intake remains a leading cause of morbidity and mortality in children under five [1, 2]. Despite global efforts, 2024 data report that 150.2 million children (23.2%) are stunted—mainly in Asia (51%) and Africa (43%)—and 42.8 million are wasted, including 12.2 million (1.9%) who are severely wasted [4]. Although SAM mortality has declined, many children still fail to recover fully [5]. The Sphere Standards recommend a recovery rate above 75% [6], but this target remains unmet in many developing countries [7, 26, 27], including Ethiopia [28]. This systematic review and meta‐analysis aimed to estimate the pooled recovery rate of under‐five children from severe acute malnutrition (SAM) in Ethiopia and compare it with international benchmarks and findings from other countries.

In this study, the pooled recovery rate was found to be 71.37% (95% CI: 67.57–75.16%), based on a random‐effects model, indicating that nearly three out of four children recover from SAM in the Ethiopian context. However, this figure falls short of the Sphere Standard, which sets an acceptable recovery threshold at above 75% [6]. Despite notable global improvements in case fatality rates for severe acute malnutrition (SAM), which vary widely from 22% to 90% depending on context [5], recovery outcomes remain inconsistent. The recovery rate observed in this study falls slightly below the internationally recommended target of 75%, indicating persistent gaps in the quality, coverage, and consistency of SAM treatment programs in Ethiopia. These findings suggest an urgent need to strengthen program implementation, including improving therapeutic feeding protocols, and enhancing health system capacity to deliver sustained and equitable care. Ethiopia's current recovery rate aligns closely with the sub‐Saharan African regional average of 71.2% [11], indicating shared structural and operational challenges within health systems across the region. However, when compared to country‐specific programmatic outcomes, Ethiopia's performance appears suboptimal. For instance, Nigeria has reported a markedly higher recovery rate of 95.7% among children aged 6–59 months with complicated SAM [12], which may be attributed to the implementation of more robust clinical protocols and better‐resourced therapeutic programs. Similarly, India's adoption of community‐based management of acute malnutrition has yielded a recovery rate of 78% [9], emphasizing the efficacy of decentralized, community‐oriented service delivery models in enhancing treatment outcomes. Conversely, Ethiopia's pooled recovery rate is substantially higher than the 46.4% reported in a multicenter study conducted across South Asia [10], and also exceeds Pakistan's recovery rate of 68.6% [8]. These differences may reflect variations in program coverage, health facility quality, socioeconomic conditions, and caregiver engagement. The current recovery rate is consistent with previous national estimates, which have ranged between 70% and 72% [13, 14, 15], suggesting a relatively stable yet persistently suboptimal performance in SAM management over recent years. This recovery rate falls below the national standard for severe acute malnutrition management, which sets a target recovery rate of over 75% [81]. The substantial heterogeneity among the included studies (I² = 98%) reflects significant variability in treatment outcomes across regions and settings in Ethiopia. This may be attributed to differences in health infrastructure, treatment protocols, and contextual factors such as sociocultural practices, food insecurity [23], and caregiver education. These findings underscore the need for context‐specific strategies within a more standardized national framework for SAM management.

In this study, clear regional disparities were observed. The highest recovery rates were reported in the southern regions of Ethiopia—Harari (83.1%), Somali (81.7%), Dire Dawa (79.8%), Oromia (75.8%), and SNNP (73.4%). These regions may benefit from relatively better access to healthcare services, more effective implementation of the Management of Acute Malnutrition (MAM) strategy, and more stable sociopolitical conditions. In contrast, the lowest recovery rates were found in the northern regions, including Afar (61.0%), Tigray (63.1%), Benishangul‐Gumuz (65.6%), and Amhara (68.1%). These lower rates may be due to internal conflicts in northern Ethiopia, which have disrupted healthcare, displaced communities, and restricted access to therapeutic supplies and transportation, along with socioeconomic, sociocultural, and food security challenges [82, 83, 84]. Recovery rates improved over time, rising from 68.5% (2015–2018) to 72.1% (2019–2025), likely due to better SAM management, training, and service expansion. However, rates remain below the SPHERE standard (≥ 75%), highlighting the need for further progress. The higher recovery rate observed in retrospective studies (72%) compared to prospective studies (68.3%), and in studies with larger sample sizes (> 420 participants, 72.9%), may reflect the benefits of larger samples. Larger studies often capture a wider range of cases and treatment settings, improving case detection and reducing bias. This leads to findings that more accurately reflect real‐world outcomes in routine healthcare contexts. The lower recovery rate among children recovering within a median duration of less than 1 month (68.1%) compared to those taking longer (77.3%) may suggest that extended treatment supports better adherence and program flexibility. The higher recovery in uncomplicated SAM cases (80.4%) likely reflects lower clinical severity, stronger caregiver support, health literacy, and follow‐up.

Children with tuberculosis, HIV/AIDS, pneumonia, malaria, and anemia experienced slower recovery from severe acute malnutrition. This finding is consistent with previous studies [16, 17, 18, 19, 20, 21]. Early identification and treatment of these comorbidities are crucial to breaking the undernutrition–disease cycle, reducing mortality, and improving recovery outcomes [85]. Children with diarrhea, vomiting, shock, and nasogastric (NG) tubes had a longer recovery time than those without these conditions. A previous study in Ethiopia also reported that children with diarrhea had prolonged recovery from severe acute malnutrition [21]. Similarly, a study in Bangladesh found that children with severe acute malnutrition experienced diarrhea primarily due to infections, particularly Shigella‐induced dysentery [86]. Severe diarrhea and vomiting can lead to dehydration, which in turn results in serum electrolyte imbalances [87]. Identifying the underlying cause of diarrhea and treating it appropriately is essential to accelerate the recovery of children. Vitamin A supplementation, ready‐to‐use therapeutic food, deworming, and antibiotics such as amoxicillin have been shown to improve recovery rates in children with severe acute malnutrition. These findings are consistent with previous studies [22, 24, 25]. Therefore, these interventions are beneficial for the nutritional recovery of children.

## 5 Limitations and Strengths of the Study

Most included studies were retrospective, which limited their ability to adjust for confounding factors due to the lack of socio‐demographic data. High heterogeneity and publication bias were additional limitations. The high heterogeneity likely resulted from differences in study design, participant characteristics, healthcare infrastructure, and socioeconomic disparities. In some regions, the limited number of studies with small sample sizes may have affected the accuracy of the results. Nevertheless, this review provides updated evidence on SAM recovery in Ethiopia, with most studies conducted after 2020. Sensitivity analysis showed that no single study disproportionately influenced the overall pooled prevalence, supporting the robustness and reliability of the findings. A thorough search of reputable journals was conducted, and all relevant analyses were performed to estimate the pooled recovery rate and its associated factors. Each analysis included at least three studies to ensure adequate sample size and precise estimates. These findings can inform policymakers, stakeholders, NGOs, and health workers working to improve recovery outcomes among children under five with severe acute malnutrition.

## 6 Conclusion

This systematic review and meta‐analysis found that the overall recovery rate of under‐five children from severe acute malnutrition (SAM) in Ethiopia remains below the minimum international SPHERE standard of 75%. The Ethiopian Ministry of Health and partner NGOs should strengthen the integrated management of comorbidities by ensuring routine screening and treatment for tuberculosis, HIV, pneumonia, malaria, anemia, and dehydration symptoms (diarrhea, vomiting, shock) within SAM protocols. Consistent supply of essential treatments—such as ready‐to‐use therapeutic food (RUTF), vitamin A, amoxicillin, and deworming—must be prioritized in all facilities.

## Author Contributions

**Temesgen Gebeyehu Wondmeneh:** conceptualization, investigation, funding acquisition, writing – original draft, methodology, visualization, validation, writing – review and editing, resources, supervision, data curation, software, formal analysis, project administration. **Oumer Abdulkadir Ebrahim:** conceptualization, investigation, funding acquisition, writing – original draft, writing – review and editing, visualization, validation, methodology, software, formal analysis, project administration, data curation, supervision, resources.

## Funding

The authors received no specific funding for this work.

## Conflicts of Interest

The authors declare no conflicts of interest.

## Transparency Statement

The lead author Temesgen Gebeyehu Wondmeneh affirms that this manuscript is an honest, accurate, and transparent account of the study being reported; that no important aspects of the study have been omitted; and that any discrepancies from the study as planned (and, if relevant, registered) have been explained.

## Supporting information

Supporting file 1.DOCX.

Supporting file 2.docx.

Supporting file 3.docx.

Supporting file 4.docx.

Supporting file 1. do file.

## Data Availability

All data generated or analyzed during this study are included in this article.
