# Supplementary material for: Recovery Rate of Under‐Five Children From Severe Acute Malnutrition and Its Predictors in Ethiopia: A Systematic Review and Meta‐Analysis
Source: Health Sci Rep. 2026 Jan 26;9(2):e71788. doi: 10.1002/hsr2.71788 (PMC12834707; doi:10.1002/hsr2.71788)
Supplement: Supplementary file 2 — Supporting file 2.docx. [file HSR2-9-e71788-s005.docx]

Supplementary file 2. Comprehensive search strategy for recovery of children from severe acute malnutrition in Ethiopia

| Databases | Key search terms or phrases |
| --- | --- |
| **Web of science** | Title contains **Recovery** AND Title contains **Children** AND Title contains exact phrase **Severe acute malnutrition** AND Title contains **Ethiopia** |
| Limited by | research article, English language and publication date 01/01/2015 to 23/7/2025 |
| Total articles | 30 |
| **Scopus** | Recovery "children "severe acute malnutrition" Ethiopia |
| Limited by | research articles, open access, and publication year from 2015 to 2025 |
| Total articles | 26 |
| **PubMed** | (((((Recovery) AND ((ffrft[Filter])) AND ((Recovery) AND ((ffrft[Filter]) AND (2015/1/1:2025/7/23[pdat])))) AND (((Child[MeSH Terms]) OR (Child)) OR (Children) AND ((ffrft[Filter]) AND (2015/1/1:2025/7/23[pdat])))) AND (((("Severe Acute Malnutrition"[MeSH Terms]) OR ("Severe Acute Malnutrition")) OR ("Acute Malnutrition Severe")) OR ("Malnutrition Severe Acute") AND ((ffrft[Filter]) AND (2015/1/1:2025/7/23[pdat])))) AND ((Ethiopia) OR (Ethiopia[MeSH Terms]) AND ((ffrft[Filter]) AND (2015/1/1:2025/7/23[pdat]))) |
| Total articles | 66 |
| **Google scholar** | "recovery rate " AND "children" AND "severe acute malnutrition" AND Ethiopia |
| Total articles | 540 |
| **African Journals Online** | "Recovery rate" 'children" "severe acute malnutrition" Ethiopia |
| Total articles | 15 |
| **Grand total** | 677 |
