# Supplementary material for: Recovery Rate of Under‐Five Children From Severe Acute Malnutrition and Its Predictors in Ethiopia: A Systematic Review and Meta‐Analysis
Source: Health Sci Rep. 2026 Jan 26;9(2):e71788. doi: 10.1002/hsr2.71788 (PMC12834707; doi:10.1002/hsr2.71788)
Supplement: Supplementary file 3 — Supporting file 3.docx. [file HSR2-9-e71788-s001.docx]

| **Supplementary file 3 Extracted data for the recovery rate of children from severe acute malnutrition in Ethiopia** | | | | | | | |  |  |
| --- | --- | --- | --- | --- | --- | --- | --- | --- | --- |
| Study ID | Author | Publication year | region | Study design | Sample size | Recovered | Percentage | Name of data extractor | Date of data extracted |
|  | Budul AB, et al (40). | 2020 | Somalia | Retrospective | 350 | 286 | 81.7% | TGW&OAE | 15/3/2024 |
|  | Akeberegn A, et al(41) | 2023 | SNNP | Retrospective | 241 | 192 | 79.7% | TGW&OAE | 15/3/2024 |
|  | Negussie AS, et al (42) | 2020 | Addis Ababa | Retrospective | 304 | 214 | 70.4% | TGW&OAE | 15/3/2024 |
|  | Baraki AG, et al (38) | 2020 | Amhara | Retrospective | 1690 | 1050 | 62.13% | TGW&OAE | 15/3/2024 |
|  | Bekalu A, et al(23) | 2022 | Oromia | prospective | 423 | 327 | 77.3% | TGW&OAE | 15/3/2024 |
|  | Wondim A, et al (43) | 2020 | Benishangul Gumuz | Retrospective | 398 | 262 | 65.8% | TGW&OAE | 15/3/2024 |
|  | Fikrie A, et al (44) | 2019 | SNNP | Retrospective | 381 | 272 | 69.3% | TGW&OAE | 15/3/2024 |
|  | Abebe A, et al(45) | 2023 | SNNP | prospective | 476 | 340 | 71.4% | TGW&OAE | 15/3/2024 |
|  | Tsegaye A, et al (7) | 2022 | Oromia | Retrospective | 357 | 284 | 79.6% | TGW&OAE | 15/3/2024 |
|  | Tegegne AS, et al(46) | 2021 | Afar | Retrospective | 650 | 408 | 62.9% | TGW&OAE | 15/3/2024 |
|  | Derseh, B, et al (19) | 2018 | Amhara | Retrospective | 413 | 231 | 55.9% | TGW&OAE | 15/3/2024 |
|  | Lencha B, et al (47) | 2023 | Oromia | Retrospective | 763 | 711 | 93.2% | TGW&OAE | 15/3/2024 |
|  | Atnafe B, et al (48) | 2019 | Dire Dawa | Retrospective | 713 | 569 | 79.8% | TGW&OAE | 15/3/2024 |
|  | Abate BB, et al (49) | 2020 | Amhara | Retrospective | 600 | 390 | 65% | TGW&OAE | 15/3/2024 |
|  | Asres DT, et al (50) | 2018 | Amhara | Retrospective | 401 | 208 | 51.9% | TGW&OAE | 15/3/2024 |
|  | Gebremichael DY (51) | 2015 | SNNP | Retrospective | 420 | 346 | 82.4% | TGW&OAE | 16/3/2024 |
|  | Gebrezgi D, et al (52) | 2019 | Amhara | Retrospective | 401 | 208 | 51.9% | TGW&OAE | 16/3/2024 |
|  | Wagnew F, et al (53) | 2019 | Amhara | Retrospective | 416 | 288 | 69.2% | TGW&OAE | 16/3/2024 |
|  | Bizuneh FK, et al (54) | 2022 | Benishangul Gumuz | Retrospective | 454 | 297 | 65.4% | TGW&OAE | 16/3/2024 |
|  | Yadeta SK, et al (55) | 2024 | Oromia | Retrospective | 402 | 360 | 89.6% | TGW&OAE | 16/3/2024 |
|  | F Adem, et al(37) | 2020 | Oromia | Retrospective | 133 | 34 | 25.6% | TGW&OAE | 16/3/2024 |
|  | Kidane GF, et al (56) | 2023 | Tigray | prospective | 232 | 176 | 75.9% | TGW&OAE | 16/3/2024 |
|  | Teshome G, et al (57) | 2019 | SNNP | prospective | 216 | 172 | 79.6% | TGW&OAE | 16/3/2024 |
|  | Mekuria G, et al (58) | 2017 | Amhara | prospective | 253 | 197 | 77.9% | TGW&OAE | 16/3/2024 |
|  | Kitesa GY, et al (59) | 2023 | Oromia | Retrospective | 590 | 471 | 79.8% | TGW&OAE | 16/3/2024 |
|  | Desyibelew HD, et al (60) | 2017 | Amhara | Retrospective | 401 | 234 | 58.4% | TGW&OAE | 16/3/2024 |
|  | Gebremedhin K, et al (61) | 2020 | SNNP | Retrospective | 402 | 283 | 70.4% | TGW&OAE | 16/3/2024 |
|  | Adimasu M, et al (62) | 2020 | Addis Ababa | Retrospective | 423 | 344 | 81.3% | TGW&OAE | 16/3/2024 |
|  | Mengesha MM, et al (63) | 2016 | SNNP | Retrospective | 348 | 274 | 78.75 | TGW&OAE | 16/3/2024 |
|  | Kabalo MY, et al (64) | 2017 | SNNP | Retrospective | 794 | 504 | 64.9% | TGW&OAE | 16/3/2024 |
|  | Shanka NA, et al (65) | 2015 | SNNP | Retrospective | 771 | 522 | 67.7% | TGW&OAE | 17/3/2024 |
|  | Kabthymer RH, et al (66) | 2020 | Oromia | Retrospective | 375 | 274 | 73.15 | TGW&OAE | 17/3/2024 |
|  | Hassen SL, et al (67) | 2019 | Amhara | Retrospective | 406 | 306 | 75.4% | TGW&OAE | 17/3/2024 |
|  | Eyi SE, et al (68) | 2022 | Oromia | Retrospective | 486 | 334 | 68.7% | TGW&OAE | 17/3/2024 |
|  | Husen S, et al (69) | 2022 | Oromia | Retrospective | 1004 | 913 | 90.9% | TGW&OAE | 17/3/2024 |
|  | Tefera TK, et al (70) | 2020 | Amhara | Retrospective | 341 | 254 | 74.5% | TGW&OAE | 17/3/2024 |
|  | Tesfay W, et al (71) | 2020 | Tigray | prospective | 564 | 316 | 56% | TGW&OAE | 17/3/2024 |
|  | Mamo WN, et al (72) | 2019 | Amhara | Retrospective | 389 | 254 | 65.3% | TGW&OAE | 17/3/2024 |
|  | Simachew Y, et al (73) | 2020 | SNNP | prospective | 554 | 390 | 70.4% | TGW&OAE | 17/3/2024 |
|  | Bitew ZW, et al (74) | 2020 | Addis Ababa | Retrospective | 515 | 407 | 79% | TGW&OAE | 17/3/2024 |
|  | Bitew ZW, et al (75) | 2021 | Addis Ababa | Retrospective | 610 | 466 | 74.6% | TGW&OAE | 17/3/2024 |
|  | Wondmeneh TG,et al(76) | 2025 | Afar | Retrospective | 372 | 216 | 58.1% | TGW&OAE | 15/01/2025 |
|  | Workie H.M,et al(39). | 2025 | Amhara | Retrospective | 389 | 372 | 95.9% | TGW&OAE | 23/72025 |
|  | Meseret F,et at (77). | 2024 | Harar | Retrospective | 349 | 290 | 83.1% | TGW&OAE | 23/72025 |
|  | Getahun GK, et al (78). | 2024 | Addis Ababa | Retrospective | 461 | 222 | 48.2% | TGW&OAE | 23/72025 |
|  | Mekonnen GB,et al (79). | 2025 | Amhara | Retrospective | 209 | 157 | 75.1% | TGW&OAE | 23/72025 |
|  | Feleke FW, et al (80). | 2024 | Amhara | Prospective | 352 | 263 | 74.7% | TGW&OAE | 23/72025 |

TGW:Temesgen Gebeyehu Wondmeneh, OAE: Oumer Abdulkadir Ebrahim
