# Supplementary material for: Recovery Rate of Under‐Five Children From Severe Acute Malnutrition and Its Predictors in Ethiopia: A Systematic Review and Meta‐Analysis
Source: Health Sci Rep. 2026 Jan 26;9(2):e71788. doi: 10.1002/hsr2.71788 (PMC12834707; doi:10.1002/hsr2.71788)
Supplement: Supplementary file 4 — Supporting file 4.docx. [file HSR2-9-e71788-s003.docx]

Supplementary file 4. Summary of quality assessments for included studies

| Author, publication year | Selection | | | | Comparability | Outcome | | | Score | Quality study |
| --- | --- | --- | --- | --- | --- | --- | --- | --- | --- | --- |
|  | Representativeness | Selection of non-exposed cohort | Ascertainment of exposure | outcome was not present at start of study | Controlling confounding | Assessment of outcome | long enough follow up for the occurrence of outcomes | Adequacy of follow-up of cohorts |  |  |
| Budul AB, et al (40). 2020 | 1 | 1 | 1 | 1 | 2 | 1 | 1 | 1 | 9/9 | Good |
| Akeberegn A, et al (41). 2023 | 1 | 1 | 1 | 1 | 2 | 1 | 1 | 1 | 9/9 | Good |
| Negussie AS, et al (42). 2020 | 0 | 1 | 1 | 1 | 2 | 1 | 1 | 1 | 8/9 | Good |
| Baraki AG, et al (38). 2020 | 1 | 1 | 1 | 1 | 2 | 1 | 1 | 1 | 9/9 | Good |
| Bekalu A, et al (23). 2022 | 1 | 1 | 1 | 1 | 2 | 1 | 1 | 1 | 9/9 | Good |
| Wondim A, et al (43). 2020 | 1 | 1 | 1 | 1 | 2 | 1 | 1 | 1 | 9/9 | Good |
| Fikrie A, et al (44). 2019 | 1 | 1 | 1 | 1 | 2 | 1 | 1 | 1 | 9/9 | Good |
| Abebe A, et al (45). 2023 | 0 | 1 | 1 | 1 | 2 | 1 | 1 | 1 | 8/9 | Good |
| Tsegaye A, et al (7). 2022 | 1 | 1 | 1 | 1 | 2 | 1 | 1 | 1 | 9/9 | Good |
| Tegegne AS, et al (46). 2021 | 1 | 1 | 1 | 1 | 2 | 1 | 1 | 1 | 9/9 | Good |
| Derseh, B, et al (19). 2018 | 0 | 1 | 1 | 1 | 2 | 1 | 1 | 1 | 8/9 | Good |
| Lencha B, et al (47). 2023 | 1 | 1 | 1 | 1 | 2 | 1 | 1 | 1 | 9/9 | Good |
| Atnafe B, et al (48). 2019 | 1 | 1 | 1 | 1 | 2 | 1 | 1 | 1 | 9/9 | Good |
| Abate BB, et al (49). 2020 | 1 | 1 | 1 | 1 | 2 | 1 | 1 | 1 | 9/9 | Good |
| Asres DT, et al (50). 2018 | 0 | 1 | 1 | 1 | 2 | 1 | 1 | 1 | 8/9 | Good |
| Gebremichael DY (51). 2015 | 1 | 1 | 1 | 1 | 2 | 1 | 1 | 1 | 9/9 | Good |
| Gebrezgi D, et al (52). 2019 | 1 | 1 | 1 | 1 | 0 | 1 | 1 | 1 | 7/9 | Good |
| Wagnew F, et al (53). 2019 | 1 | 1 | 1 | 1 | 2 | 1 | 1 | 1 | 9/9 | Good |
| Bizuneh FK, et al (54). 2022 | 1 | 1 | 1 | 1 | 2 | 1 | 1 | 1 | 9/9 | Good |
| Yadeta SK, et al (55). 2024 | 1 | 1 | 1 | 1 | 2 | 1 | 1 | 1 | 9/9 | Good |
| F Adem, et al (37). 2020 | 0 | 1 | 1 | 1 | 2 | 1 | 0 | 1 | 7/9 | Good |
| Kidane GF, et al (56). 2023 | 0 | 1 | 1 | 1 | 2 | 1 | 1 | 1 | 8/9 | Good |
| Teshome G, et al (57). 2019 | 0 | 1 | 1 | 1 | 2 | 1 | 0 | 1 | 7/9 | Good |
| Mekuria G, et al (58). 2017 | 0 | 1 | 1 | 1 | 2 | 1 | 1 | 0 | 7/9 | Good |
| Kitesa GY, et al (59). 2023 | 1 | 1 | 1 | 1 | 2 | 1 | 1 | 1 | 9/9 | Good |
| Desyibelew HD, et al (60). 2017 | 1 | 1 | 1 | 1 | 2 | 1 | 1 | 1 | 9/9 | Good |
| Gebremedhin K, et al (61). 2020 | 1 | 1 | 1 | 1 | 2 | 1 | 1 | 1 | 9/9 | Good |
| Adimasu M, et al (62). 2020 | 1 | 1 | 1 | 1 | 2 | 1 | 1 | 1 | 9/9 | Good |
| Mengesha MM, et al (63). 2016 | 1 | 1 | 1 | 1 | 2 | 1 | 1 | 1 | 9/9 | Good |
| Kabalo MY, et al (64). 2017 | 1 | 1 | 1 | 1 | 2 | 1 | 1 | 1 | 9/9 | Good |
| Shanka NA, et al(65). 2015 | 1 | 1 | 1 | 1 | 2 | 1 | 1 | 1 | 9/9 | Good |
| Kabthymer RH, et al (66). 2020 | 1 | 1 | 1 | 1 | 2 | 1 | 1 | 1 | 9/9 | Good |
| Hassen SL, et al (67). 2019 | 1 | 1 | 1 | 1 | 2 | 1 | 1 | 1 | 9/9 | Good |
| Eyi SE, et al (68). 2022 | 1 | 1 | 1 | 1 | 2 | 1 | 1 | 1 | 9/9 | Good |
| Husen S, et al (69). 2022 | 1 | 1 | 1 | 1 | 2 | 1 | 1 | 1 | 9/9 | Good |
| Tefera TK, et al (70). 2020 | 1 | 1 | 1 | 1 | 2 | 1 | 1 | 1 | 9/9 | Good |
| Tesfay W, et al (71). 2020 | 1 | 1 | 1 | 1 | 0 | 1 | 1 | 1 | 7/9 | Good |
| Mamo WN, et al (72). 2019 | 1 | 1 | 1 | 1 | 2 | 1 | 1 | 1 | 9/9 | Good |
| Simachew Y, et al (73). 2020 | 1 | 1 | 1 | 1 | 2 | 1 | 1 | 1 | 9/9 | Good |
| Bitew ZW, et al (74). 2020 | 1 | 1 | 1 | 1 | 2 | 1 | 1 | 1 | 9/9 | Good |
| Bitew ZW, et al (75). 2021 | 1 | 1 | 1 | 1 | 0 | 1 | 1 | 1 | 7/9 | Good |
| Wondmeneh TG, et al (76). 2025 | 1 | 1 | 1 | 1 | 2 | 1 | 1 | 1 | 9/9 | Good |
| Workie H.M,et al(39). 2025 | 1 | 1 | 1 | 1 | 2 | 1 | 1 | 1 | 9/9 | Good |
| Meseret F,et at (77). 2024 | 1 | 1 | 1 | 1 | 2 | 1 | 1 | 1 | 9/9 | Good |
| Getahun GK, et al (78). 2024 | 1 | 1 | 1 | 1 | 2 | 1 | 1 | 1 | 9/9 | Good |
| Mekonnen GB,et al (79). 2025 | 0 | 1 | 1 | 1 | 2 | 1 | 1 | 1 | 8/9 | Good |
| Feleke FW, et al (80). 2024 | 1 | 1 | 1 | 1 | 2 | 1 | 1 | 1 | 9/9 | Good |

**Newcastle-Ottawa Quality Assessment Form for Cohort Studies**

Note: A study can be given a maximum of one star for each numbered item within the Selection and Outcome categories. A maximum of two stars can be given for Comparability.

**Selection**

1. Representativeness of the exposed cohort
2. Truly representative (one star)
3. Somewhat representative (one star)
4. Selected group
5. No description of the derivation of the cohort
6. Selection of the non-exposed cohort
7. Drawn from the same community as the exposed cohort (one star)
8. Drawn from a different source
9. No description of the derivation of the non-exposed cohort
10. Ascertainment of exposure
11. Secure record (e.g., surgical record) (one star)
12. Structured interview (one star)
13. Written self-report
14. No description
15. Other
16. Demonstration that outcome of interest was not present at start of study
17. Yes (one star)
18. No

**Comparability**

1. Comparability of cohorts on the basis of the design or analysis controlled for confounders
2. The study controls for age, sex and marital status (one star)
3. Study controls for other factors (list) _________________________________ (one star)
4. Cohorts are not comparable on the basis of the design or analysis controlled for confounders

**Outcome**

- 1. Assessment of outcome

1. Independent blind assessment (one star)
2. Record linkage (one star)
3. Self-report
4. No description
5. Other
   1. Was follow-up long enough for outcomes to occur
6. Yes (one star)
7. No

Indicate the median duration of follow-up and a brief rationale for the assessment above:____________________

- 1. Adequacy of follow-up of cohorts

1. Complete follow up- all subject accounted for (one star)
2. Subjects lost to follow up unlikely to introduce bias- number lost less than or equal to 20% or description of those lost suggested no different from those followed. (one star)
3. Follow up rate less than 80% and no description of those lost
4. No statement

Thresholds for converting the Newcastle-Ottawa scales to AHRQ standards (good, fair, and poor):

Good quality: 3 or 4 stars in selection domain AND 1 or 2 stars in comparability domain AND 2 or 3 stars in outcome/exposure domain

Fair quality: 2 stars in selection domain AND 1 or 2 stars in comparability domain AND 2 or 3 stars in outcome/exposure domain

Poor quality: 0 or 1 star in selection domain OR 0 stars in comparability domain OR 0 or 1 stars in outcome/exposure domain
